# The unfolded protein response influences therapy outcome and disease progression in chronic lymphocytic leukaemia

**DOI:** 10.1038/s41598-025-13495-1

**Published:** 2025-07-28

**Authors:** Umair Tahir Khan, Kim Clarke, Gina Eagle, Melanie Oates, Peter Hillmen, Sandrine Jayne, Martin J. S. Dyer, Alex Phipps, Francesco Falciani, Rosalind E. Jenkins, Andrew R Pettitt

**Affiliations:** 1 Department of Molecular and Clinical Cancer Medicine, Institute of Systems, Molecular and Integrative Biology, University of Liverpool, Liverpool, UK; 2 Computational Biology Facility, University of Liverpool, Liverpool, UK; 3 Leeds Cancer Centre, Leeds Teaching Hospitals Trust, Leeds, UK; 4 Leicester Cancer Research Centre, The Ernest and Helen Scott Haematological Research Institute, University of Leicester, Leicester, UK; 5 PRED Innovation Centre Welwyn, Roche Products, Shire Park, Welwyn Garden City, Herts, UK; 6 Department Pharmacology and Therapeutics, Institute of Systems, Molecular and Integrative Biology, CDSS Bioanalytical Facility, Liverpool Shared Research Facilities, University of Liverpool, Liverpool, UK

**Keywords:** Proteomics, CLL cells, Drug resistance and disease progression, eIF2 signalling, PERK linked to resistant phenotype, Cancer immunotherapy, Cancer therapeutic resistance

## Abstract

Since genomics, epigenomics and transcriptomics have provided only a partial explanation of chronic lymphocytic leukaemia (CLL) heterogeneity, and since concordance between mRNA and protein expression is incomplete, we related the CLL proteome to clinical outcome. CLL samples from patients who received fludarabine-containing chemoimmunotherapy were analysed by mass spectrometry (SWATH-MS). One dataset compared pre-treatment samples associated with an optimal versus suboptimal response, while another compared paired samples collected before treatment and at disease progression. eIF2 signalling (pivotal to the unfolded protein response (UPR)), was identified as the most enriched pathway in both datasets (respective z-scores: − 6.245 and 3.317; *p* < 0.0001), as well as in a fludarabine-resistant CLL cell line established from HG3 cells (z-score: − 2.121; *p* < 0.0001). Western blotting revealed that fludarabine-resistant HG3 cells expressed higher levels of PERK, which phosphorylates the regulatory eIF2α subunit, and lower levels of BiP, an HSP70 molecular chaperone that inactivates PERK but preferentially binds to misfolded proteins during ER stress. The PERK inhibitor, GSK2606414, sensitised resistant, but not sensitive, HG-3 cells to fludarabine without affecting background cell viability or cytotoxicity induced by the BCL-2 inhibitor venetoclax. These findings identify the UPR as a novel determinant of therapy outcome and disease progression in CLL.

**Supplementary Information:**

The online version contains supplementary material available at 10.1038/s41598-025-13495-1.

## Introduction

Chronic lymphocytic leukaemia (CLL) is a malignancy of mature CD5^+^ B lymphocytes that accumulate in the blood, bone and secondary lymphoid tissues. It accounts for approximately 40% of all adult leukaemias, making it the most common form of leukaemia in Western countries^1^. The disease is notable for its heterogeneous clinical course and response to treatment. At the start of this study, the standard first-line treatment for young, fit patients was the chemoimmunotherapy regimen fludarabine, cyclophosphamide and rituximab (FCR)^2,3^. Despite the overall effectiveness of FCR, a minority of patients do not respond and most eventually relapse^4^. Such relapses are due to the emergence of treatment-resistant clones leading to disease progression and acquired drug resistance^5^.

Advances in genomics and transcriptomics have vastly improved our knowledge of CLL biology. Despite this, the biological basis of CLL heterogeneity and variable therapy outcome remains incompletely understood. One potential explanation for this knowledge gap is that the relationship between genome, transcriptome and proteome is not strictly linear. Indeed, it is estimated that the correlation between mRNA and protein expression may be as low as 30%.^6^ There are several potential reasons for this discordance. First, when mRNA levels fall in response to an external stimulus, proteins with a long half-life may continue to be present at high levels^7^. Second, even in steady state, protein levels may be regulated independently of mRNA levels through protein degradation and the effect of RNA binding proteins, non-coding RNAs and miRNAs on protein synthesis^8,9^. To add to the complexity, the relationship between mRNA and protein expression may be influenced by genetic polymorphisms that vary between individuals.

Given that the cellular phenotype is ultimately determined by gene expression at the protein level, we postulated that characterising the CLL proteome and relating it to therapy outcome might provide new insights into CLL heterogeneity. By applying global cellular mass spectrometry to high-quality CLL samples obtained from a well-defined cohort of patients receiving initial therapy with fludarabine-containing chemoimmunotherapy, we identified heterogeneity in the unfolded protein response which was shown to be functionally important in a bespoke and carefully validated cell-line model.

## Results

### Patient characteristics

**Fig. 1:**
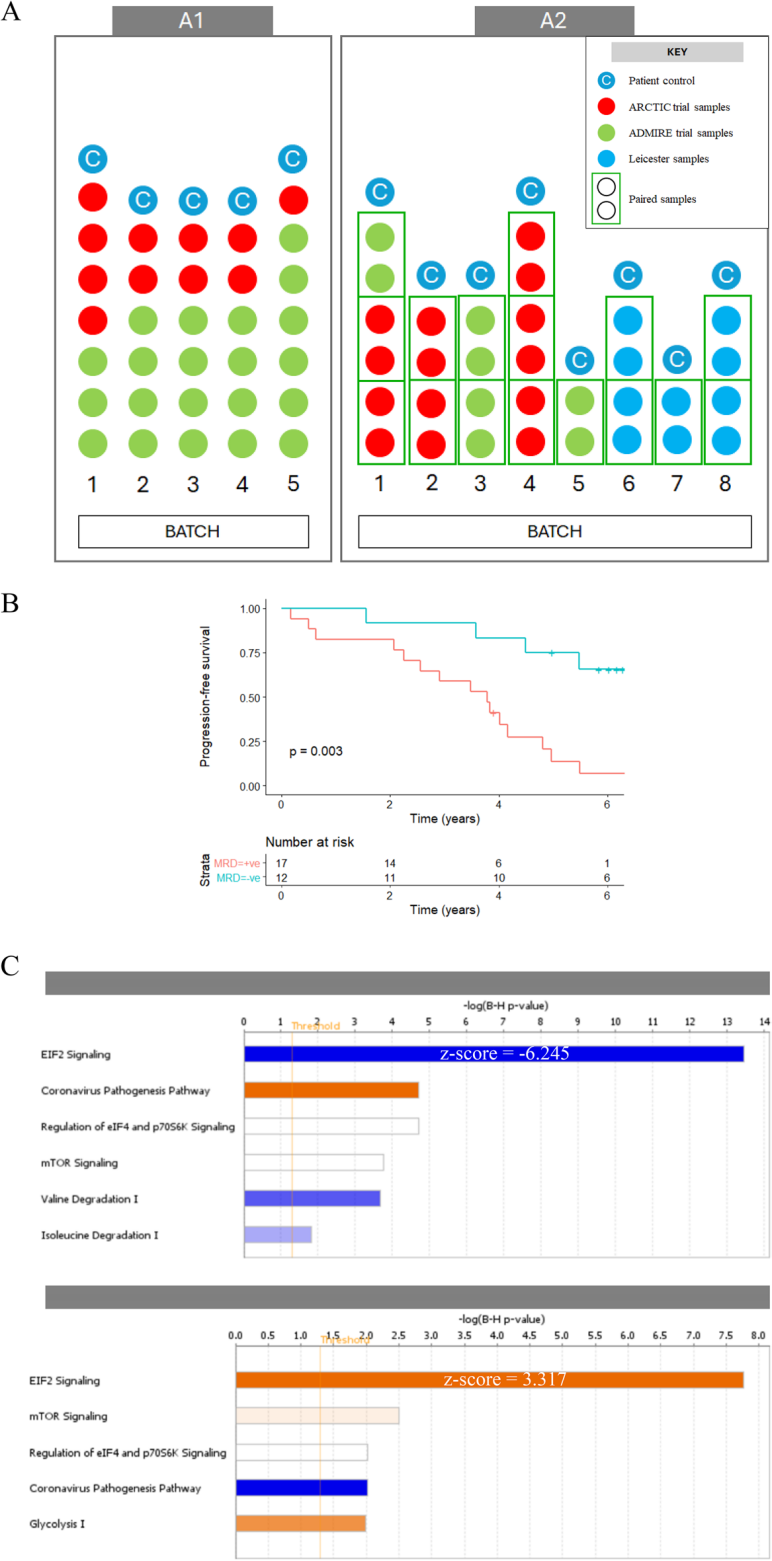
Characterisation of CLL patient cohorts. **A** Experimental design used to generate the A1 and A2 datasets. The 32 CLL samples used for the A1 dataset were tested in five batches (left panel) whereas the 32 paired CLL samples from 16 patients were tested in eight batches (right panel). Pairs of samples (pre-treatment and disease progression, denoted by rectangle around two samples) from individual patients were processed together in the same batch. Samples labelled as ‘C’ represent control sample from the same patient. The samples have been coloured coded to represent samples from different trials. **B** Kaplan-Meier plot comparing the progression-free survival (PFS; time to progression or death) of patients who achieved optimal (MRD–; blue line) or suboptimal (MRD+; red line) responses in the A1 dataset. **C** Ingenuity Pathway Analysis of the A1 and A2 datasets revealed EIF2 signalling as the most enriched pathway in both. A Benjamini-Hochberg correction was applied to the pathways identified to control for false discovery.

The study employed 64 samples from 48 CLL patients undergoing initial treatment with FCR with or without mitoxantrone. Patients were grouped into two separate cohorts based on the comparative approach used to generate the proteomic datasets. The first approach (A1) compared pre-treatment samples from 32 patients who achieved optimal versus suboptimal cytoreduction based on the detection of measurable residual disease (MRD) in the bone marrow three months after completing treatment using cut-off of one CLL cell in 10^4^ leukocytes (Fig. 1A). The second approach (A2) compared paired samples obtained from 16 patients before treatment and at disease progression (Fig. 1A). Patient characteristics relating to the A1 and A2 datasets are summarised in Table 1. The median age was 64 (IQR 59-68.5) and 70 (IQR 63.8–73.5), respectively, and there was a preponderance of males (75% and 67%, respectively). Within the A1 dataset, there were no statistically significant differences in pre-treatment variables between MRD + and MRD– groups, although a higher proportion of MRD + patients had progressive stage A and B (Binet) disease before start of treatment compared to MRD- patients (65% vs. 25%, *p* = 0.06, Fisher Exact statistic). As expected, progression-free survival (PFS) was shorter in patients who were MRD + at the end of treatment compared with those who were MRD– (*p* = 0.003; Fig. 1B). In the A2 dataset, the median time to progression was 43 months (30.6–51.2).

**Table 1:** Baseline patient characteristics for A1 and A2 datasets.

		Stratified by MRD			Overall
	Overall	Positive	Negative		
n	32	17	12	n	16
Age [median (IQR)]	64(59-68.5)	66 (64-69)	64 (59.5-66.5)	Age [median (IQR)]	70 (63.8-73.5)
Sex (%)				Sex (%)	
Female	7 (21.9)	4 (23.5)	2 (16.7)	Female	4 (25.0)
Male	24 (75.0)	13 (76.5)	10 (83.3)	Male	8 (50.0)
NA	1 (3.1)	0 (0.0)	0 (0.0)	NA	4 (25.0)
Stage (%)				Stage (%)	
A	3 (9.4)	3 (17.6)	0 (0.0)	A	2 (12.5)
B	11 (34.4)	8 (47.1)	3 (25.0)	B	4 (25.0)
C	17 (53.1)	6 (35.3)	9 (75.0)	C	6 (37.5)
NA	1 (3.1)	0 (0.0)	0 (0.0)	NA	4 (25.0)
iwCLL response (%)				iwCLL response (%)	
CR	16 (50.0)	6 (35.3)	9 (75.0)	CR	5 (31.2)
Non-CR	15 (46.9)	11 (64.7)	3 (25.0)	Non-CR	7 (43.8)
NA	1 (3.1)	0 (0.0)	0 (0.0)	NA	4 (25.0)
Treatment (%)				Time to relapse in months [median (IQR)]	43.0 (30.6-51.2)
FCM-miniR	6 (18.8)	2 (11.8)	4 (33.3)	Relapse (%)	
FCR	14 (43.8)	7 (41.2)	6 (50.0)	< 3 years	8 (50.0)
FCRM	11 (34.4)	8 (47.1)	2 (16.7)	> 3 years	4 (25.0)
NA	1 (3.1)	0 (0.0)	0 (0.0)	NA	4 (25.0)
IGHV status (%)				Treatment (%)	
Mutated	7 (21.9)	2 (11.8)	4 (33.3)	FC	1 (6.2)
Unmutated	11 (34.4)	7 (41.2)	4 (33.3)	FCR	10 (62.5)
NA	1 (3.1)	0 (0.0)	0 (0.0)	FCRM	1 (6.2)
TP53 mutation/17p deletion (%)				NA	4 (25.0)
Absent	11 (34.4)	5 (29.4)	5 (41.7)	IGHV status (%)	
Present	3 (9.4)	1 (5.9)	2 (16.7)	Mutated	2 (12.5)
NA	18 (56.2)	11 (64.7)	5 (41.7	Unmutated	7 (43.8)
				NA	7 (43.8
				TP53 mutation/17p deletion (%)	
				Absent	6 (37.5)
				Present	2 (12.5)
				NA	8 (50.0)

All patients met the IwCLL criteria for starting initial therapy, and all demographic, clinical and molecular data relate to the pre-treatment timepoint. Some patients received Mitoxantrone in addition to FCR, with rituximab at either standard (FCRM; AdMIRe trial) or reduced (FCM-miniR; ARCTIC trial) dose. IGHV: Immunoglobulin heavy-chain variable region gene; CR: complete remission; NA: not available; MRD: measurable residual disease; CR: complete remission; NA: not available; IQR: interquartile range.

### Characterisation of the CLL proteome

The experimental design used to generate the A1 and A2 datasets is shown in Fig. 1A. In the A1 dataset, 4696 proteins were quantified across 32 test CLL samples and 5 controls, whereas in the A2 dataset, 3567 proteins were quantified across 32 test CLL samples and 8 controls. As expected, levels of less abundant proteins with lower mean peak areas varied more widely^10^ than levels of highly abundant proteins (Supplementary Figure S1). Principal component analysis (PCA) was utilised as a graphical representation tool to assess the variance between samples and batches, and the efficacy of the batch correction protocol (Supplementary Figures S2 and S3). Once batch corrected, the data showed significant heterogeneity among individual CLL samples but no clear clustering between MRD + and MRD– samples in the A1 dataset, or between pre-treatment and progression samples in the A2 dataset (Supplementary Figure S4).

### Correlation of eIF2 signalling with therapy outcome

Given no clear clustering at the global proteome level was seen, it was hypothesised that a subset of proteins is likely to correlate with therapy outcome. Subsequently, DPE analysis identified 528 and 464 altered proteins (*p* < 0.05) in the A1 and A2 datasets, respectively, and these were interrogated using Ingenuity Pathway Analysis (IPA: Illumina, California, USA). Canonical Pathway analysis revealed that the top pathway identified in the A1 dataset was eukaryotic Initiation Factor-2 (eIF2) signalling. This pathway was predicted to be downregulated in samples from MRD + patients with a highly significant Benjamini-Hochberg p-value (*p* < 0.0001, z-score − 6.245; Fig. 1C). The same pathway was also the most highly enriched in the A2 dataset, with upregulation predicted in samples obtained at disease progression (*p* < 0.0001, z-score 3.317; Fig. 1C).

### Generation and validation of a cell-line model

**Fig. 2:**
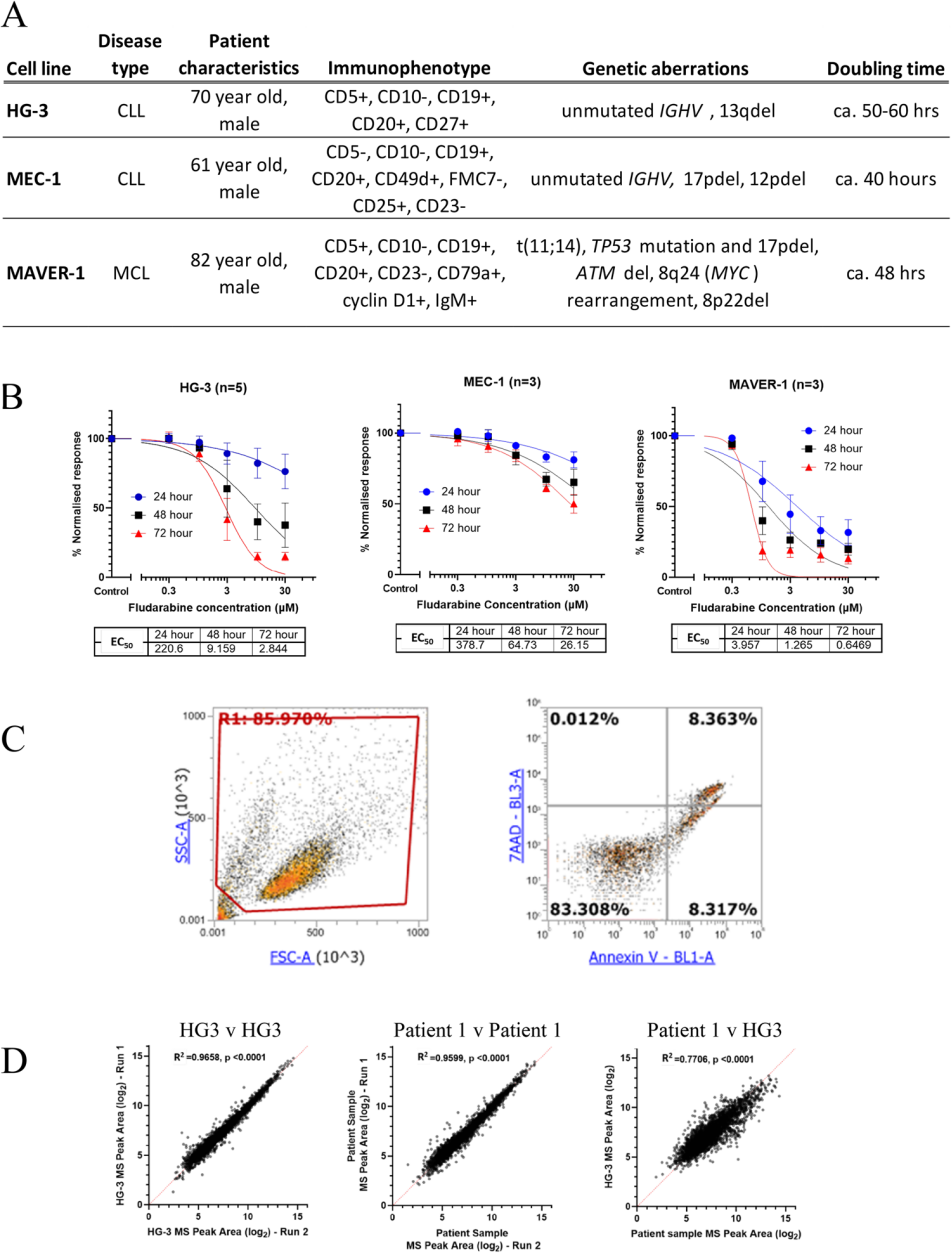
Selection of a cell line model to investigate fludarabine resistance. **A** Summary of cell line characteristics for HG-3, MEC-1 and MAVER-1. The information has been summarised from multiple sources, including DSMZ (https://www.dsmz.de/) and Cellosaurus (https://www.cellosaurus.org/). **B** Fludarabine dose-response curves for the three parental cell lines. Fludarabine at 0, 0.3, 1, 3, 10 and 30µM was added to HG-3, MEC-1 and MAVER-1 cells. Viability was measured at 24, 48 and 72 h and normalised relative to that of untreated control cells at the same timepoint. **C** Representative scatterplot showing the flow cytometry gating strategy used to measure cell viability. Forward and side scatter characteristics were used to identify intact cells (left panel). Cells were separated by quadrants into live (annexin–/7-AAD–), early apoptotic (annexin+, 7-AAD–) and late apoptotic (annexin+/7-AAD+) (right panel). (**D**) Representative scatter plot showing the correlation in total protein expression between HG3 v HG3 cells, patient sample v patient sample and HG3 cells v patient sample.

To explore the potential role of eIF2 signalling as a determinant of therapy response, we sought to generate a resistant cell line using the drug induction method that reproduced at least some of the proteomic features of the A1 or A2 datasets. Owing to the complexity of treating cell lines with appropriate concentrations of multiple drugs in combination, resistance was induced to fludarabine alone on the grounds that it is the pharmacological backbone of the FCR regimen^11^. Three candidate parental cell lines (HG-3, MEC-1, MAVER-1; Fig. 2A) were assessed for their sensitivity to fludarabine over a range of drug concentrations and exposure times. MEC-1 cells were highly resistant to fludarabine and therefore considered unsuitable, whereas HG-3 and MAVER-1 cells produced full dose-response curves at 72 h with clinically relevant EC_50_ values (Fig. 2B). Representative scatterplots showing the flow cytometry gating strategy used to measure cell viability are shown in Fig. 2C. The HG-3 cell line was chosen in preference to MAVER-1 on the grounds that it is derived from CLL, whereas MAVER-1 originates from mantle-cell lymphoma. The suitability of the HG-3 cell line was further corroborated by the close resemblance of its overall proteomic signature to that of primary CLL cells (R^2^ > 0.75, *p* < 0.0001; Fig. 2D).

**Fig. 3:**
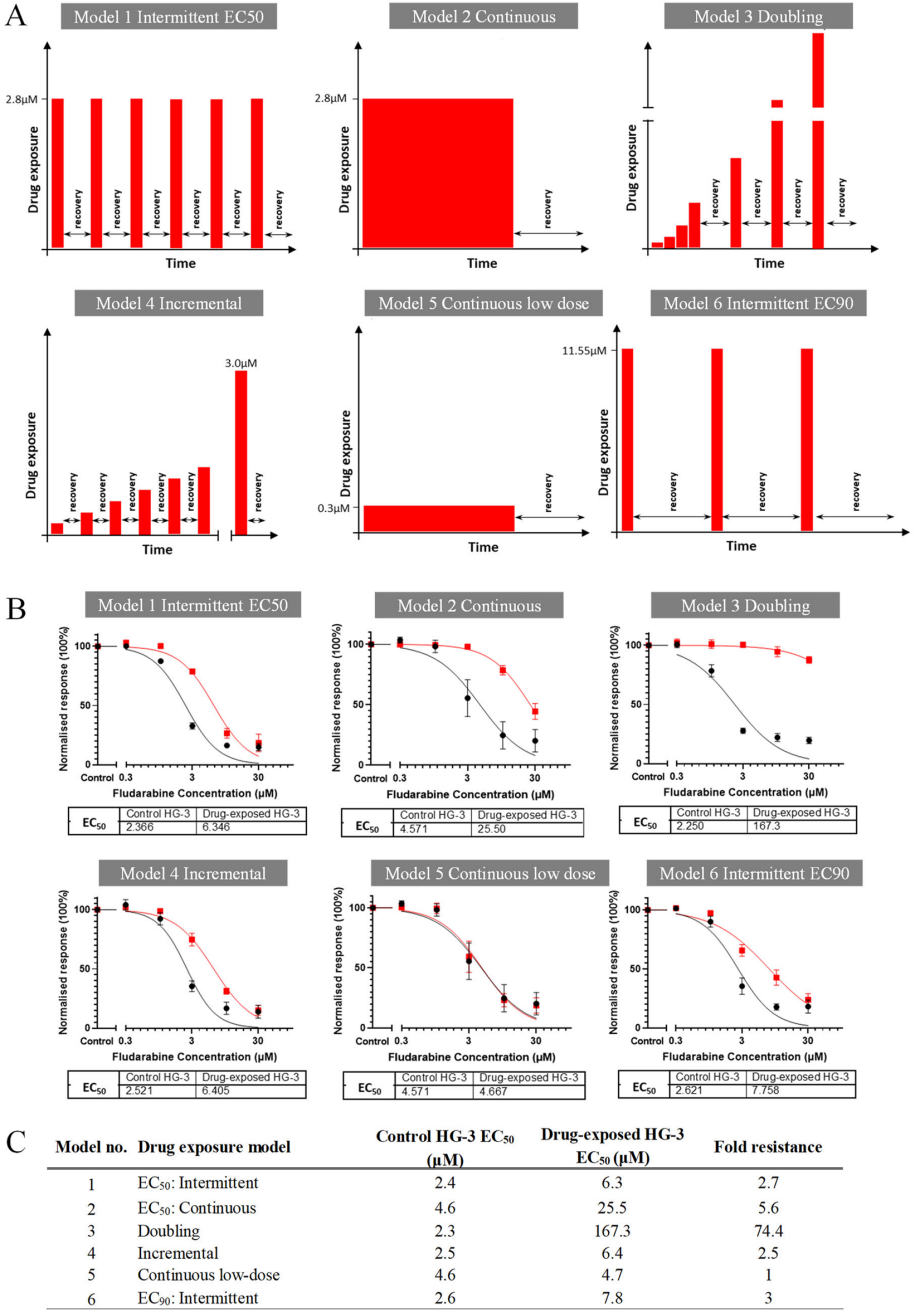

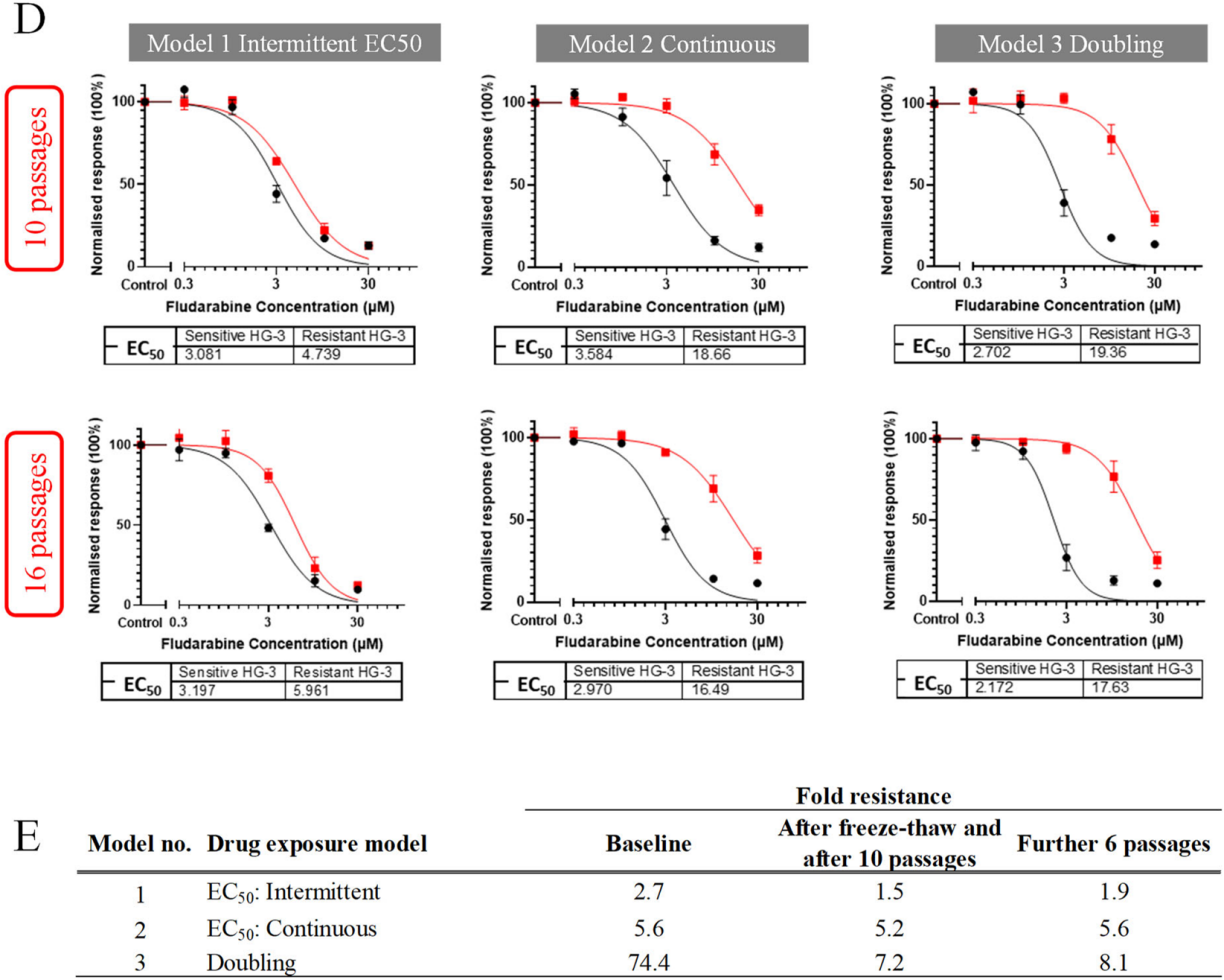
Drug exposure models to develop a cell line model of fludarabine resistance. **A** Graphical representations of the different drug induction models used to generate fludarabine-resistant HG-3 cell lines. For each model, cells were exposed to fludarabine or 0.1% DMSO (vehicle control) in parallel. Resistance was achieved in 100 to 120 days in most models except number 5 which remained fully sensitive. **B** Fludarabine dose-response curves for HG-3 cells resulting from different drug exposure strategies. Curves for cells exposed to fludarabine and DMSO are shown as red and black, respectively. Cell viability was measured using the annexin V/7-AAD assay after incubating the derived cell lines with fludarabine or 0.1% DMSO for 72 h. Viability was normalised relative to the DMSO control and EC_50 (72 h)_ values (in µM) calculated for each model. **C** Fold resistance values for different drug exposure models. Values were calculated by dividing the fludarabine EC_50 (72 h)_ of drug-exposed HG-3 with the fludarabine EC_50 (72 h)_ of the corresponding DMSO-exposed (control) cells. **D** Stability of fludarabine-resistant phenotype in the three selected cell-line models after 10 and 16 passages. Dose-response experiments were performed at baseline, after 10 passages (*n* = 3) and after six further passages (*n* = 2). EC_50 (72 h)_ values (in µM) are shown for each set of experiments. Vehicle control HG-3 cells are sensitive (black line), whereas fludarabine-exposed HG-3 are shown to maintain a level of resistance over multiple passages (red line). **E** Fold-resistance values are shown at baseline and after undergoing either 10 or 16 additional passages in the absence of fludarabine.

To maximise the likelihood of generating a suitable fludarabine-resistant cell line, six different drug-exposure models were employed based on published methodology^12^ and the cyclical dosing schedule employed for fludarabine-based regimens (Fig. 3A). Fludarabine resistance was acquired in five of the six models (Fig. 3B and C), three of which (models 1–3) were selected for further investigation on the grounds that they were generated by different drug exposure strategies and produced different fold-resistance (FR) values: model 1 (intermittent EC_50_ model, FR 2.5); model 2 (continuous EC_50_ model, FR 5.6); and model 3 (doubling exposure model, FR 74.4). To assess the stability of these models, sensitivity to fludarabine was re-assessed after 10 and 16 additional passages in the absence of drug exposure. FR values remained constant in model 2 but fell significantly after 10 passages in model 1 (1.5) and model 3 (7.2) after which they stabilised (Fig. 3D and E).

**Fig. 4:**
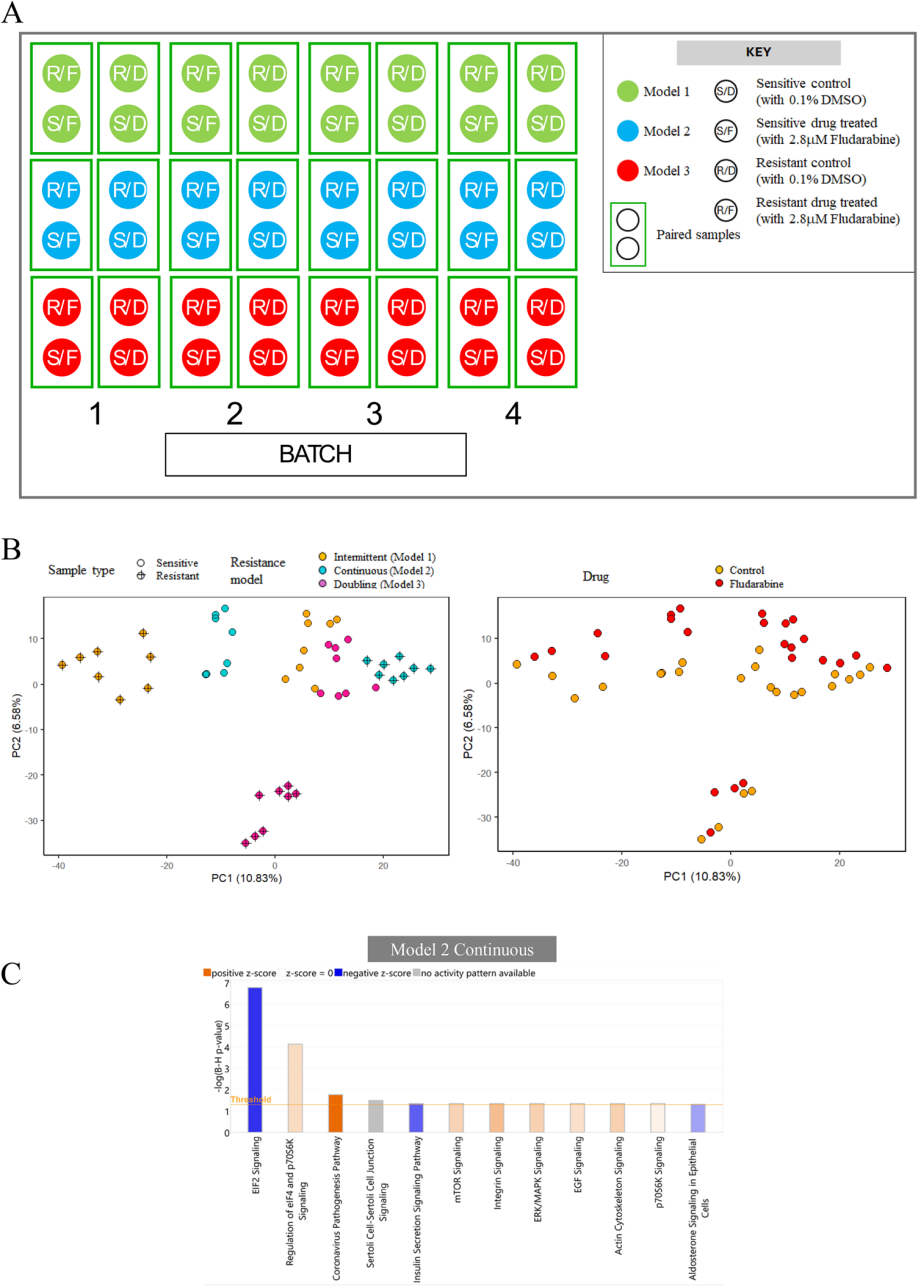
Proteomic analysis of fludarabine-resistant cell-line models (models 1–3). **A** Sensitive (S) and resistant (R) cells from each model were exposed to 0.1% DMSO (D) or drug (fludarabine, 2.8µM (F)) for 24 h. The viability of the cells measured by Annexin V and 7-AAD was on average *≥* 77%. Each group of four samples in each of the three models was tested together in the same batch of 12 samples, and the experiment was replicated over four batches comprising 48 samples in total. **B** Principal component analysis of proteomic data from all 48 HG-3 samples, with batch-corrected data coded for resistance model and phenotype (left panel) and batch-corrected data coded for treatment with fludarabine or DMSO (control) (right panel). **C** Canonical pathway analysis in IPA revealed that EIF2 signalling was the most enriched pathway in model 2 (continuous exposure). A Benjamini-Hochberg correction was applied to the pathways identified to reduce the false positive pathways identified.

Cell-line models 1–3 were then analysed by SWATH-MS before and after fludarabine treatment (2.8 µM for 24 h), allowing visualisation of not only steady-state protein expression but also fludarabine-induced changes prior to the onset of extensive cytotoxicity. The experimental design is shown in Fig. 4A. In total, 4015 proteins were quantified across 48 samples. After correcting for batch effects, the samples clustered based on the cell-line model, whether the cell-line was fludarabine sensitive or resistant and, in the case of fludarabine-sensitive cell lines, whether or not the cells had been exposed to fludarabine (Fig. 4B).

For each of the three cell-line models, proteins differentially expressed in untreated fludarabine-sensitive versus -resistant cells (*p* < 0.05), or in fludarabine-sensitive cells treated with fludarabine or DMSO control (*p* < 0.05), were analysed by IPA. Notably, eIF2 signalling was the most enriched (upregulated) pathway in fludarabine-resistant cells in model 2 (Benjamini-Hochberg Pvalue < 0.0001, z-score − 2.121; Fig. 4C) These findings replicated the pathway analysis of the A1 proteomic dataset and, in doing so, validated the model 2 cell line as a suitable model for FCR resistance. eIF2 was also the most enriched (upregulated) pathway induced by fludarabine in sensitive model 2 cells (Benjamini-Hochberg Pvalue < 0.0001, z-score 4.651, data not shown).

### Identification of PERK as a key determinant of fludarabine sensitivity/resistance

**Fig. 5:**
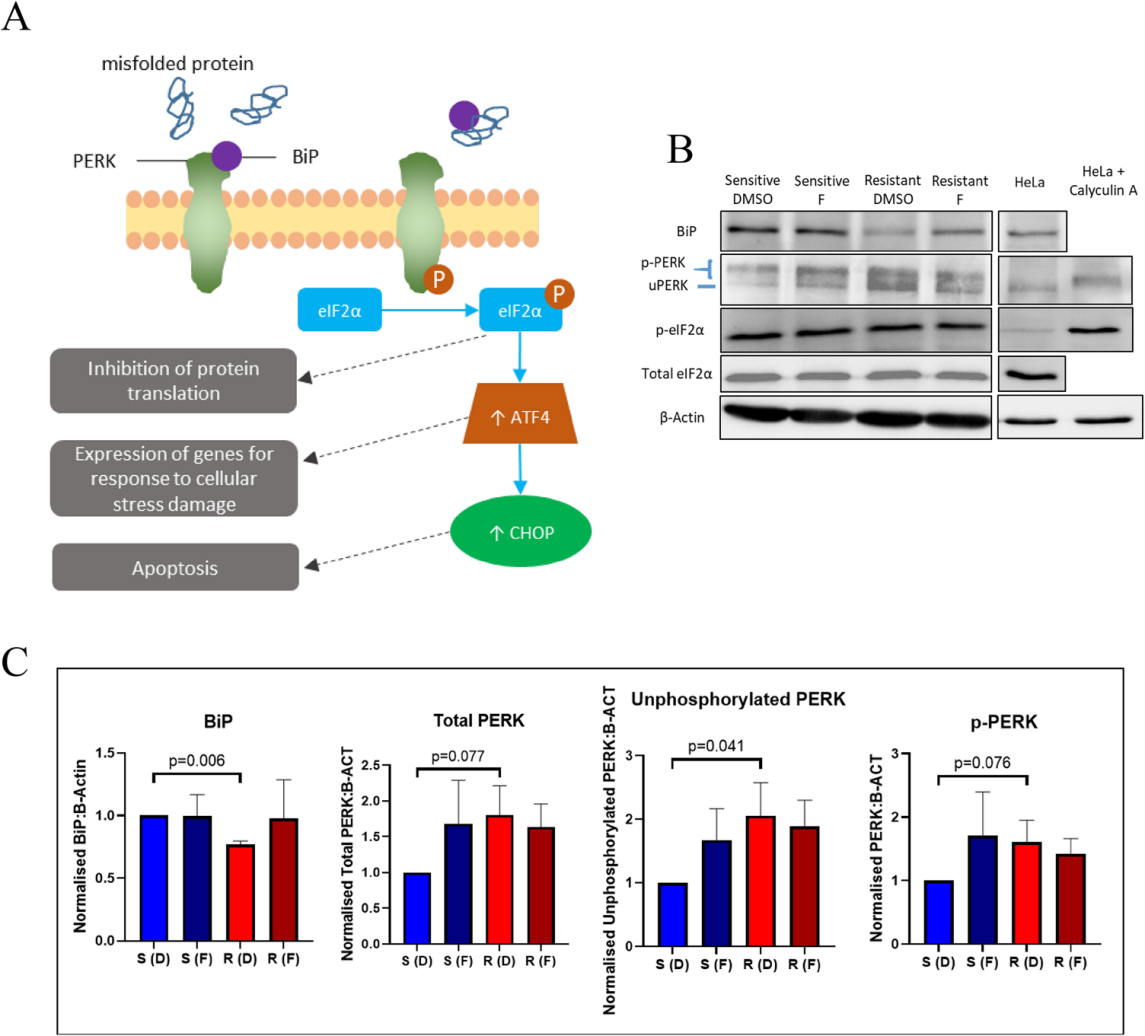
Measurement of key components of eIF2 signalling pathway. **A** Diagram of the eIF2α signalling pathway. During cellular stress, BiP binds to misfolded proteins and dissociates from PERK leading to PERK autophosphorylation and activation. Phosphorylated PERK (p-PERK) phosphorylates eIF2α resulting in global reduction of protein synthesis and increased translation of ATF4 (both of which alleviate ER stress) and, depending on the context, increased ATF4-dependent transcription of CHOP (which induces apoptosis). **B** Representative western blot showing levels of BiP, p-PERK, unphosphorylated PERK (uPERK), phosphorylated eIF2α (p-eIF2α), total eIF2α and β-actin in sensitive and resistant model 2 HG-3 cells following 24 h exposure to 3µM fludarabine or DMSO control. Untreated HeLa cells were used as a positive control for BiP, uPERK and total eIF2α and a negative control for p-PERK and p-eIF2α, whereas HeLa cells treated with calyculin A (a phosphatase inhibitor) were used as a positive control for p-PERK and p-eIF2α. The uncropped images are shown in Supplementary Fig. 5. **C** Bar charts showing levels of BiP and PERK relative to β-actin as measured by densitometry (*n* = 3). Statistical significance was assessed using the ratio paired t-test. S: sensitive cells; R: resistant cells; D: DMSO treated; F: fludarabine treated.

To investigate key components of the eIF2 pathway (Fig. 5A) in model 2 cell-line, resistant and sensitive model 2 cells were analysed by western blotting. Levels of the following proteins were assessed: eIF2α, the regulatory subunit of the eIF2 complex; PRKR-like endoplasmic reticulum kinase (PERK), which phosphorylates eIF2α when it is in its autophosphorylated/activated form; and endoplasmic reticulum chaperone binding immunoglobulin protein (BiP), which binds to PERK and inhibits autophosphorylation/activation except under conditions of ER stress when it preferentially binds to misfolded proteins (Fig. 5B and C). Levels of total and phosphorylated eIF2α were similar in fludarabine-sensitive and -resistant model 2 cells. However, the resistant cells had lower levels of BiP (*p* = 0.006) and higher levels of phosphorylated, unphosphorylated and total PERK (*p* = 0.076, 0.04 and 0.077, respectively). These findings identified PERK as a potential determinant of fludarabine cytotoxicity in the model 2 cell-line.

**Fig. 6:**
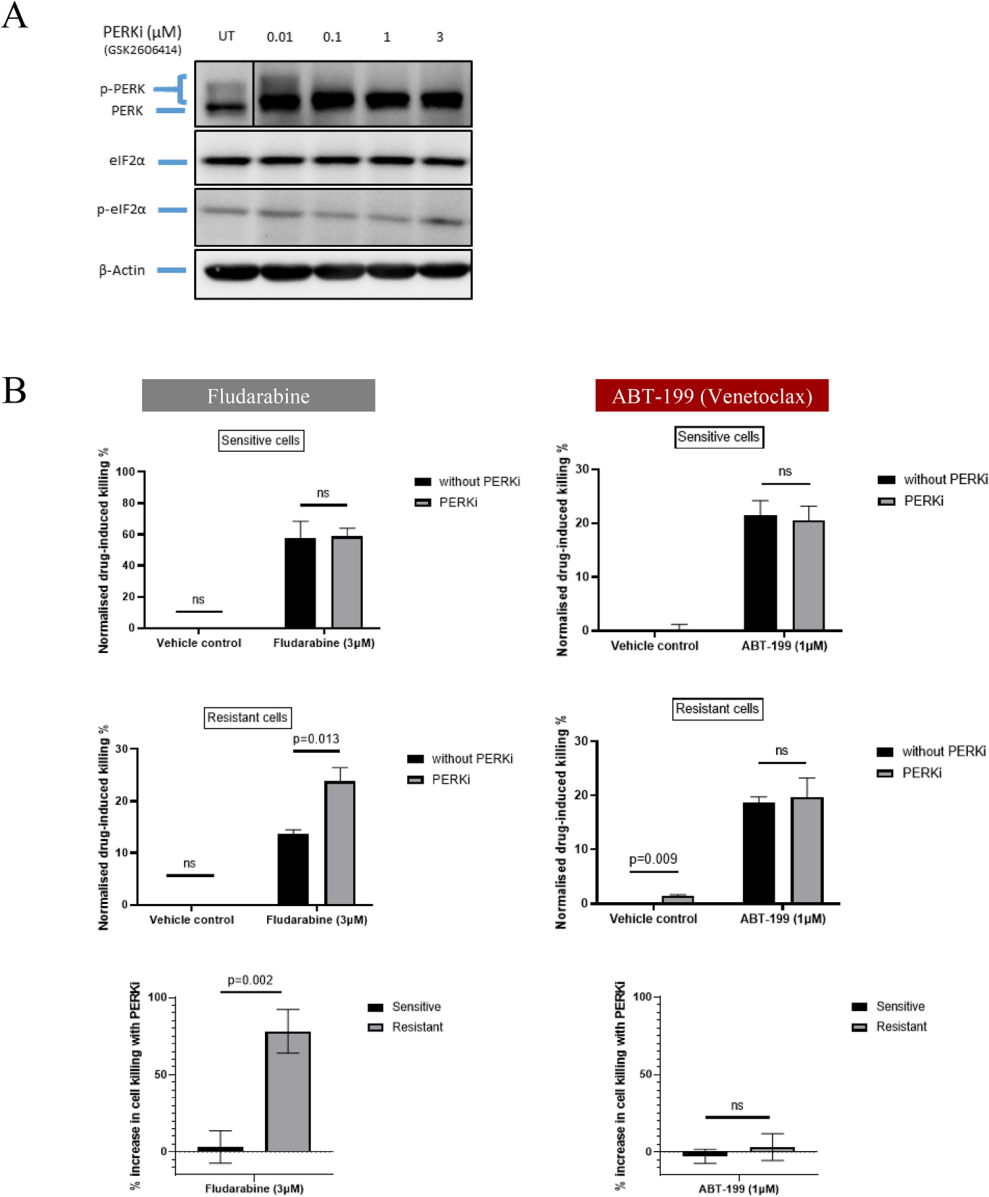
Effect of inhibition of PERK on the drug-induced killing of fludarabine-sensitive and resistant model 2 HG-3 cells. Cells were incubated with 1µM GSK2606414 for six hours before adding fludarabine (3µM), venetoclax (ABT-199, 1µM) or DMSO control (0.1%). Due to the short half-life of GSK2606414, the drug was replenished every 24 h to maintain its inhibitory effect. **A** Western blot showing the effect of PERK inhibition measured at six hours. UT: untreated (0.1% DMSO). A box was put around UT condition for PERK blot as other concentrations were cropped out. **B** Fludarabine-induced killing of sensitive and resistant model 2 cells in the presence or absence of GSK2606414 for 72 h (left panel). Venetoclax-induced killing of sensitive model 2 cells in the presence or absence of GSK2606414 for 72 h (right panel). The percentage increase in cell killing of the resistant and sensitive cells lines following PERK inhibition are also shown for fludarabine and Venetoclax (bottom graphs) All experiments were conducted in biological triplicates. Statistical significance was assessed using the paired or unpaired t-test, as appropriate. ns: non-significant.

To test this idea, we examined the effect of GSK2606414, a specific inhibitor of PERK autophosphorylation^13,14^. At a concentration that produced complete loss of phosphorylated PERK (1µM; Fig. 6A), GSK2606414 increased the fludarabine-induced killing of resistant model 2 cells by 74% (*p* = 0.013) without having any appreciable effect on the fludarabine-induced killing of sensitive model 2 cells, the killing of fludarabine-sensitive or -resistant model 2 cells by venetoclax (an inhibitor of Bcl-2 that induces apoptosis directly by activating the mitochondrial death pathway), or the viability of either cell type in the absence of drug exposure (Fig. 6B). These findings confirmed that PERK was not only overexpressed in fludarabine-resistant model 2 cells but also contributed in a direct and specific way to their drug-resistant phenotype. Given the close similarity between the proteomic signature of fludarabine resistance in the model 2 cell-line and that associated with suboptimal response to FCR in the A1 dataset, it is reasonable to speculate that PERK may also be functionally important in influencing clinical outcome.

## Discussion

This study employed a global cellular proteomics approach to discover new aspects of CLL heterogeneity. It exploited the availability of high-quality, clinically annotated CLL samples obtained from a well-defined cohort of patients receiving initial chemoimmunotherapy with FCR. The samples were analysed by SWATH-MS in carefully designed experiments that included control samples and batch correction. Comparison of pre-treatment samples from patients who did or did not achieve MRD negativity, and of paired samples obtained before treatment and at disease progression, revealed eIF2 signalling as the most enriched pathway. It was also the most enriched pathway in a cell-line model of fludarabine resistance, further characterisation of which showed altered levels of PERK (regulates eIF2α) and BiP (regulates PERK). The functional contribution of PERK to fludarabine resistance (and by implication suboptimal response to FCR) was confirmed by showing that resistance was partially reversed in a highly selective way by the specific PERK inhibitor, GSK2606414. Our findings are novel as neither PERK nor the eIF2 pathway have previously been linked to CLL heterogeneity or therapy response.

eIF2 is a heterotrimeric complex that fulfils a vital role in mRNA translation by binding initiator methionyl-tRNA (Met-tRNAi) and transferring it to the 40 S ribosomal subunit in a GTP-dependent fashion. The regulatory subunit of eIF2 (eIF2α) is phosphorylated by PERK, one of three transmembrane sensors of ER stress (the other two being ATF6 and IRE1α). Phosphorylation of eIF2α prevents the eIF2 complex exchanging GDP for GTP and thereby halts global mRNA translation, whilst also increasing the translation of specific mRNAs that alleviate ER stress such as activating transcription factor 4 (ATF4)^15,16^. In non-stressed conditions, PERK is held in an inactive (unphosphorylated) state by binding to BiP. However, in situations of ER stress, BiP preferentially binds to misfolded proteins and releases PERK. PERK then becomes activated through autophosphorylation and phosphorylates eIF2α, resulting in a reduction in the overall protein load^17^. This sequence of events, the UPR, either allows cells to survive in the face of ER stress, or actively eliminates cells that are irreparably damaged through upregulation of genes that induce apoptosis such as C/EBP homologous protein (CHOP) – a transcriptional target of ATF4^18–20^. The factors that determine whether the UPR results in cell survival or cell death are incompletely understood but are likely to include the magnitude and duration of ER stress, as well as the cellular context^16,21–24^.

The increased eIF2 signalling observed in sensitive model 2 cells following fludarabine treatment is entirely consistent with the known effect of multiple cytotoxic drugs (including purine analogues) in inducing ER stress^25^, as well as the role of ER stress in regulating spontaneous apoptosis in primary CLL cells^26^. Furthermore, the observed association between PERK over-expression and fludarabine resistance in model 2, as well as the reversal of such resistance by GSK2606414, indicates that PERK was fulfilling a cytoprotective (oncogenic) function in this setting. The idea that PERK can behave as an oncogene is in keeping with the more aggressive phenotype associated with PERK over-expression in multiple cancers^27–30^, as well as the anti-tumour effects of PERK inhibition in myeloma cells^31^. Despite the evidence supporting the use of PERK inhibition to partially reverse fludarabine resistance in this study, it is important to note that GSK2606414 is associated with pancreatic toxicity manifesting as weight loss and elevated blood glucose levels^32^ which may limit its clinical utility.

The fact that GSK2606414 had no effect on the fludarabine-induced killing of sensitive model 2 cells indicates that PERK was redundant in this setting, presumably due to activation of dominant alternative stress pathways in response to fludarabine treatment. The idea that stress pathways are “layered” and function in a sequence that depends on the stressor and cellular context is entirely in keeping with our previous demonstration that TP53 and poly(ADP-ribose) polymerase play a hierarchical role in mediating the cytotoxicity of purine analogues^33,34^.

Given that fludarabine-resistant model 2 cells expressed higher levels of PERK, the reduced eIF2 signalling observed in these cells, as well as in pre-treatment samples from CLL patients who responded sub-optimally to FCR, may seem paradoxical. However, it is important to note that fludarabine-resistant model 2 cells not only over-expressed PERK but also under-expressed BiP, which binds to and inactivates all three ER stress sensors including ATF6 and IRE1α^35^. It is therefore reasonable to speculate that ATF6 and IRE1α^35^ may have been more activated in fludarabine-resistant model 2 cells, resulting in lower levels of ER stress as a driver of eIF2 signalling. The complex role of BiP in ER stress is illustrated by the fact that the UPR can be associated with either over-expression^36–41^ or under-expression^42^ of BiP, depending on the cellular context. Furthermore, in CLL the role of BiP extends beyond ER stress to signalling required for antibody production in response to IgM stimulation^43^. A transcriptomic analysis to further evaluate the paradoxical response could be highly informative.

PERK inhibition had no effect on eIF2α phosphorylation in fludarabine-resistant model 2 cells so it is not possible to draw any conclusions about the extent to which the reduced eIF2 signalling in these cells contributed to their drug-resistant phenotype. It is, however, reasonable to infer that the cytoprotective effect of PERK in model 2 cells was mediated not by phosphorylation of eIF2α, but rather by phosphorylation of PERK’s other substrate, nuclear factor-erythroid factor 2-related factor 2 (Nrf2), which co-ordinates the cellular response to oxidative stress.

It is unclear why eIF2 signalling was increased in primary CLL samples obtained at disease progression relative to those obtained before treatment. Clonal selection by FCR seems unlikely given that eIF2 signalling was reduced, rather than increased, in pre-treatment CLL samples obtained from patients who underwent a sub-optimal response to the same treatment. A more plausible explanation is that increased eIF2 signalling is a feature of CLL subclones that seed relapse due to their higher proliferative rate. In keeping with this idea, activation of the UPR has been linked to a more aggressive form of prostate cancer^44^.

It was beyond the scope of the present study to investigate the effects of other ER stressors, such as tunicamycin, on HG3 cells. It also needs to be recognised that, despite their proteomic similarity with primary CLL cells, HG-3 cells are immortalised through EBV transformation and are therefore an imperfect model of CLL. For that matter, even primary CLL cells obtained from the blood do not recapitulate the complex interactions that take place in the in-vivo CLL microenvironment. Another limitation of the study is that it did not investigate confounding variables associated with fludarabine resistance. Having said that, the main chromosomal abnormality associated with inadequate response to FCR [del(17p)] was present in only one patient in our cohort making it difficult to draw firm conclusions.

These limitations aside, our study has identified the UPR as a novel determinant of therapy outcome and disease progression in CLL. A potential pathway to translation is provided by the favourable safety profile and encouraging preliminary efficacy of an orally administered PERK inhibitor (HC-5404) in a phase I study of heavily pretreated patients with advanced solid tumours^45^. At a more general level, our study illustrates the potential of whole-cell proteomics to discover novel aspects of cancer biology that have been overlooked by genomic and transcriptomic profiling.

## Methods

### CLL patients and clinical data

All patients included in the study were receiving initial chemoimmunotherapy that included fludarabine, cyclophosphamide and rituximab (FCR), either as part of the NCRI AdMIRe or ARCTIC trials (*n* = 43), or outside these trials at the Leicester Royal Infirmary (LRI; *n* = 5). Clinical data was obtained from the Genomics England Research Environment^46^ in the case of trial patients, and the Leicester Haematological Malignancies Tissue Bank (LHMTB) for patients treated at the LRI.

### CLL samples

CLL samples were obtained from the UK CLL Biobank^47^ in the case of the trial patients, and the LHMTB in the case of patients treated at the LRI. Additional CLL control samples were obtained from the Liverpool Blood Disease Biobank (LBDB). All methods were carried out in accordance with relevant guidelines and regulations, and informed consent was obtained from all subjects and/or their legal guardians. Whole blood was collected and processed into cryopreserved peripheral blood mononuclear cells (PBMC) in accordance with the Research Ethics Committee approval for the respective biobank (UK CLL Biobank: 14/NW/1014 and 19/NW/0573; LHMTB: 06/Q2501/122; LBDB: 16/NW/0810). Samples included in the study had > 90% B-cell purity and viability of > 60%, except in rare circumstances where sample availability was limited.

### Mass spectrometry

CLL samples were prepared for proteomic analysis by SWATH-MS as described previously^10^. Digested and desalted peptides were resuspended in 0.1% formic acid (FA), and the equivalent of 1 µg of protein from each sample was subjected to mass spectrometry using a Triple TOF 6600 (SCIEX). The peptides were injected via an Eksigent NanoLC 415 System (SCIEX) fitted with an ACQUITY UPLC Peptide BEH C18 nanoACQUITY Column (Waters, UK) and a nanoACQUITY UPLC Symmetry C18 Trap Column (Waters). The trap column was washed with 2% acetonitrile/0.1% formic acid at a rate of 2.5 µL/min for 10 min. A gradient of 2–50% acetonitrile/0.1% formic acid at a flow rate of 300nL/min over 120 min was applied to the analytical column. The mass spectrometer was operated in positive ion mode with an MS scan of 50ms and MS/MS scans of 30ms, giving a total cycle time of 3.1 s. An m/z range of 350–1250 and 100 variable SWATH acquisition windows were employed.

### Processing and analysis of datasets

The current SWATH data have been deposited to the ProteomeXchange Consortium^48^ via the PRIDE partner repository^49^ with the dataset identifier PXD058183. Peptides were identified using DIA-NN 1.7^50^ and our previously described CLL-specific spectral library (PRIDE ID PXD011330)^10^. Settings in DIA-NN included using an FDR of < 1%, and selecting ‘use neural networks’, ‘unrelated runs’, ‘RT profiling’, ‘RT-dependent’ in the cross-run normalisation tab, and ‘protein inference’ options, including ‘robust LC (high precision)’ quantification strategy. Data was normalised within DIA-NN software using MaxLFQ^51^. Normalised MS data were processed and analysed in RStudio (version 1.4.1103) using R [version 3.6.2 (2019-12-12)]^52^. Multiple R packages were utilised in analysing the data. Batch effects were visualised in accordance with previous publications^53,54^ (Supplementary Figure S2) and removed using *removeBatchEffect* function in *limma* package (Supplementary Figure S3)^55^. Detailed methods are described in the Supplementary material. Differential protein expression (DPE) analysis between groups of interest was also performed using *limma* package in R, following the user’s guide. Controls included in each sample batch were employed to visualise batch effect, which was accounted for in the DPE analysis by specifying batch as a blocking variable. This one-step statistical analysis was preferable to a two-step process in which batch-corrected data is used for DPE analysis, because the former reduces the error in the analysis^56^.

### Cell culture and treatment with fludarabine

HG-3, MEC-1 and MAVER-1^57–59^ cells (obtained from Leibniz Institute DSMZ-German Collection of Microorganisms and Cell Cultures GmbH) were grown in complete RPMI-1640 medium using a humidified incubator at 37 °C and 5% CO_2_. The cell lines were authenticated using short tandem repeat (StR) profiling and were regularly checked to confirm mycoplasma negativity in culture. Cells with a density of ~ 0.5 × 10^6^ cells/ml and in logarithmic growth phase were treated with fludarabine (2-Fluoroadenine-9-β-D-arabinofuranoside, cat. no. 2773-5 mg, Sigma-Aldrich) at varying doses ranging from 0 to 30µM in 48-well plates. Cell viability was measured at 24 h, 48 h and 72 h using fluorescein isothiocyanate (FITC) conjugated Annexin V (cat. no. 556420, BD Biosciences, UK) and 7-aminoactinomycin Dstaining (7-AAD, cat. no. 559925, BD Biosciences, UK). The proportion of live cells was determined for each drug concentration, and the data was normalised to the vehicle control to measure drug-induced killing. Dose-response curves were then plotted for each of the cell lines at the specified time points (24 h, 48 h and 72 h) and used to calculate the EC_50_ value at each timepoint using GraphPad Prism 9.

### Western blotting

Protein (20–50 µg) from each sample was loaded onto stacking gel and separated on SDS-polyacrylamide gel with a resolving gel of 7–15%. The separated proteins were transferred to a nitrocellulose membrane which was blocked with 1X TBST with 5% w/v non-fat dried milk (NFDM) or 5% BSA and probed with antibodies to BiP (1:1000, cat. no. ab21685, Abcam), p-eIF2α (1:500, cat. no. 9721 S, Cell Signaling Technology^®^, USA), eIF2α (1:1000, cat. no. 9722 S, Cell Signaling Technology^®^, USA), PERK (1:1000, cat. no. 3192 S, Cell Signaling Technology^®^, USA) or β-actin (1:10000, cat. no. ab6276, Abcam). The membrane was incubated with a secondary antibody in 1X TBST with 5% w/v NFDM [mouse anti-rabbit IgG (L27A9) horseradish peroxidase (HRP) conjugate (1:2000, cat. no. 5127 S, Cell Signaling Technology^®^, USA); or goat anti-mouse HRP conjugate (1:2000, cat. no. 41116161, Agilent Technologies LDS UK LTD)]. The membrane was then exposed to Western Lightning^®^ Plus enhanced chemiluminescence reagent (cat. no. NEL103001EA, PerkinElmer, Inc., U.S.A) and visualised immediately in a Bio-Rad Chemidoc™ Imaging System (Singapore). The images were imported into ImageJ 1.48 for densitometry of relevant protein bands.

### Ingenuity pathway analysis

The pathway analyses were performed using QIAGEN Ingenuity Pathway Analysis (IPA, version 76765844)^60^. Lists of differentially expressed proteins generated using *limma* were uploaded into IPA. Proteins from the dataset that met the *p* < 0.05 cut-off and were associated with a Canonical pathway in the Ingenuity Knowledge Base were included in the analysis. In the settings, ‘B lymphocytes’ was selected in the Tissues & Cell Lines section, ‘Human’ in the Species section, and ‘Ingenuity Knowledge Base (Genes Only)’ as the reference set. Benjamini-Hochberg Multiple Testing Correction p-values were used to identify the most significant pathways and to minimise the risk of false discovery.

### Statistical analysis

Categorical data are shown as frequencies and percentages, whereas continuous data are represented as median with interquartile range (IQR) unless specified otherwise. Fisher’s Exact test was used to calculate the statistical significance of categorical data. DPE analysis was performed using *limma* package in R, which uses a Bayes method to adjust the variance of a given gene based on the variance across all proteins and therefore moderates the t-test. Statistical methods for multiple-testing correction can be overly stringent for proteomic data due to several factors: limited technical repeats with patient samples, intrinsic variability of protein expression, and small sample sizes. In this study, we utilised fold change and p-values as a pragmatic approach to control false discovery rates^61^. The strength of correlation was calculated using the coefficient of determination (R^2^ where the fit was to a y = x axis rather than the best-fit line, using GraphPad Prism 9. Kaplan-Meier (KM) plots were generated in R, and a log-rank test was used to calculate p values. Univariable Cox proportional hazards modelling was performed to assess the impact of demographic and clinical variables on time-to-event (progression or death). For western blot densitometry analysis, a ratio-paired t-test was performed in GraphPad Prism 9.

## Supplementary Information

Below is the link to the electronic supplementary material.

Supplementary Material 1

## Data Availability

The datasets used and/or analysed during the current study available from the corresponding author on reasonable request. Proteomics data are available via ProteomeXchange with identifier PXD058183.
