# Supplementary material for: The unfolded protein response influences therapy outcome and disease progression in chronic lymphocytic leukaemia
Source: Sci Rep. 2025 Jul 28;15:27496. doi: 10.1038/s41598-025-13495-1 (PMC12304208; doi:10.1038/s41598-025-13495-1)
Supplement: Supplementary file 1 — Supplementary Material 1 [file 41598_2025_13495_MOESM1_ESM.pdf]

Supplementary material

Data processing in R

Normalised MS data was processed and analysed in RStudio (version 1.4.1103) using R [version 3.6.2 (2019-12-12)] [1]. Numerous R packages were utilised in analysing the data including *ggplot2* [2], *ggrepel* [3], *sva* [4], *limma* [5], *data.table* [6], *proBatch* [7], *dplyr* [8], *tibble* [9], *stringr* [10], *gtable* [11], *pamr* [12], *ggpubr* [13], *gridExtra* [14], *tableone* [15], *xtable* [16], *pROC* [17], *survivalROC* [18], *survival* [19], *tidyr* [20], and *survminer* [21].

Assessment of data quality and completeness at the protein level

Scatterplots relating the coefficient of variation with mean protein abundance were prepared for both A1 and A2 datasets (Supplementary Figure S1).

Visualisation of batch effects and their correction in A1 dataset

Various tools were employed to visualise batch effects in the datasets. The following describes the visualisation process for the A1 dataset. Firstly, a PCA plot was utilised as a graphical representation tool to assess the variance between samples and batches. In the A1 dataset, Supplementary **Error! Reference source not found.**S2A shows that batch one samples clustered separately from batches two to five, suggesting a systemic difference in variance and, therefore the presence of batch effects. This is also highlighted by the control sample from batch one which clustered separately from the control samples in other batches. Subsequently, batches two to five were grouped into a 'modified batch' (Supplementary **Error! Reference source not found.**B). The reason for forming the new modified batch grouping was to visualise that the main technical variation was between batch one and the rest of the batches. Furthermore, these new batch labels would form the basis of batch correction and avoid over-correction of batch effects between each batch.

Secondly, PVCA was used, which plots the weighted average proportion variance associated with different biological factors (such as MRD status, age, sex) and technical factor (batch effects) (Supplementary **Error! Reference source not found.**C). The plot highlighted that most of the observed variance was associated with the modified batch grouping as opposed to biological factors. This provided further evidence of the presence of batch effects. Thirdly, an unsupervised hierarchical cluster heat map was plotted (Supplementary **Error! Reference**

source not found.D), which showed clustering of samples within batch one compared to the other batches, even though the control samples clustered together. The analyses described above provided sufficient evidence to indicate that batch effects were present in the dataset, specifically between batch one and the other batches. Since batch effects could adversely impact the downstream analysis, a batch correction was performed prior to further visualisation of the data.

Assessing the quality of batch correction

Batch correction can be performed using various packages and tools in R. The *removeBatchEffect* function of the *limma* package [5] was employed, with samples grouped according to the modified batch (i.e. batch one versus the rest). The batch-corrected proteomic dataset was then visualised to see how effective the correction had been (Supplementary **Error! Reference source not found.**S3A-F). The PCA plot showed that the control samples from each batch now clustered together (Supplementary **Error! Reference source not found.**A) and with the aid of Supplementary **Error! Reference source not found.**3B, it can be seen that batch one no longer clusters separately from the other batches and that the overall variance within batch one is the same as that in the other batches. In keeping with the PCA plots, PVCA analysis (Supplementary **Error! Reference source not found.**S3C) showed that the variance associated with modified batch grouping is no longer the dominant source of variance. Furthermore, the clustering of batch one samples was no longer seen in the unsupervised hierarchical cluster heat map, although the clustering of the control samples was maintained (Supplementary **Error! Reference source not found.**D). This was corroborated by a correlation heat map, which showed that the correlation of overall protein expression between individual samples, as indicated by Spearman's rank correlation coefficient (r_s), was clearly evident among the five control samples and less so between samples within individual batches (Supplementary **Error! Reference source not found.**E). This observation was confirmed by violin plots showing the distribution of r_s values for individual proteins between and within different batches and among the control samples before and after batch correction (Supplementary **Error! Reference source not found.**S3F). Notably, after batch correction, the profile of correlation across different batches was almost identical to that seen within batches, while correlation among the control samples was further enhanced.

After batch correction, it was noted that sample 7310 was an outlier. There was nothing unusual about the clinical characteristics of the patient from which the sample was derived, and its outlier status was therefore attributed to anomalous quantification resulting from technical or experimental factors. The sample was removed from all subsequent analyses to avoid skewing the results. Taken together, the analyses described above indicate that batch correction using the *limma* package in R was successful in removing any obvious batch effects from the A1 dataset. Although it is possible that more subtle batch effects remain or that some genuine biological differences between samples may have been inadvertently removed, the batch-corrected dataset should be more reliable to visualise compared to the original dataset. Similar approaches were applied to A2 and cell line proteomic datasets.

Characterisation of the CLL proteome

Once batch corrected, the SWATH-MS data showed significant heterogeneity among individual CLL samples but no clear clustering between MRD+ and MRD– samples in the A1 dataset, or between pre-treatment and progression samples in the A2 dataset (Supplementary Figure S4).

Supplementary Figure Legends

Supplementary Figure S1. Assessment of data quality and completeness at the protein level. (**A**) Scatterplot relating the coefficient of variance with protein mean abundance across all 37 samples (32 CLL and 5 control) in the A1 dataset. (**B**) Scatterplot relating the coefficient of variance with protein mean abundance across all 40 samples (32 CLL and 8 control) in the A2 dataset. Data points are colour-coded based on whether the corresponding proteins have >75% or $\leq$75% missing values across all samples in the dataset. One protein with %CV >150 was excluded from A1 dataset dot-plot, and one protein with %CV >200 was excluded from the A2 dataset dot-plot to optimise data visualisation.

Supplementary Figure S2. V**isualisation of batch effects.** (**A**) PCA plot which revealed that batch one clustered separately from the other batches. (**B**) PCA plot with batches two to five grouped together into a 'modified batch'. (**C**) PVCA plot showing the weighted average proportion variance ascribed to batch effects and other biological factors such as age, stage and MRD status. (**D**) Unsupervised hierarchical cluster heat map showing clustering of samples in batch one.

Supplementary Figure S3. Data visualisation after limma batch correction of the A1 dataset. (**A**) PCA plot of batch-corrected data, showing that all five controls now cluster together (shown in red circle) and that variance associated with batch one is now similar to that in the other batches. (**B**) PCA plot with 'modified batch' grouping highlighting the similarity in variance between batch one and the other batches. (**C**) PVCA plot showing the weighted average proportion variance of batch-corrected data. The variance associated with the modified batch grouping is no longer dominant. (**D**) Unsupervised hierarchical cluster heat map of batch-corrected data showing that batch one does not cluster separately from the rest of the batches and that the controls continue to cluster together. (**E**) Correlation heat map of overall protein expression between individual samples, as indicated by Spearman's rank correlation coefficient (r_s). Stronger correlation is seen among the control samples compared with samples within batches. (**F**) Violin plot showing r_s values of individual proteins between and within batches and among control samples, before and after limma batch correction. The red arrows in A, B, D and E indicate the outlier sample.

Supplementary Figure S4. **Principle component analysis plots showing batch-corrected proteomic data from the A1 and A2 datasets.** (**A**) PCA plot of all 32 samples in the A1 dataset coded by MRD status. (**B**) PCA plot of all 32 samples in the A2 dataset coded by time obtained relative to treatment.

Supplementary Figure S5. **Uncropped Western Blot images.** The Western Blot results shown in Figure 5 of the main manuscript have been included here in their uncropped form. Probed with antibodies to (**A**) BiP, (**B**) p-PERK/uPERK, (**C**) p-eIF2α, (**D**) total eIF2α, (**E**) ß-actin (representative). In each case, the lanes contain in order: Sensitive HG3 treated with DMSO; Sensitive HG3 treated with Fludarabine; Resistant HG3 treated with DMSO; Resistant HG3 treated with Fludarabine; Untreated HeLa cells and HeLa cells plus Calyculin A.

Supplementary Figure S1

A

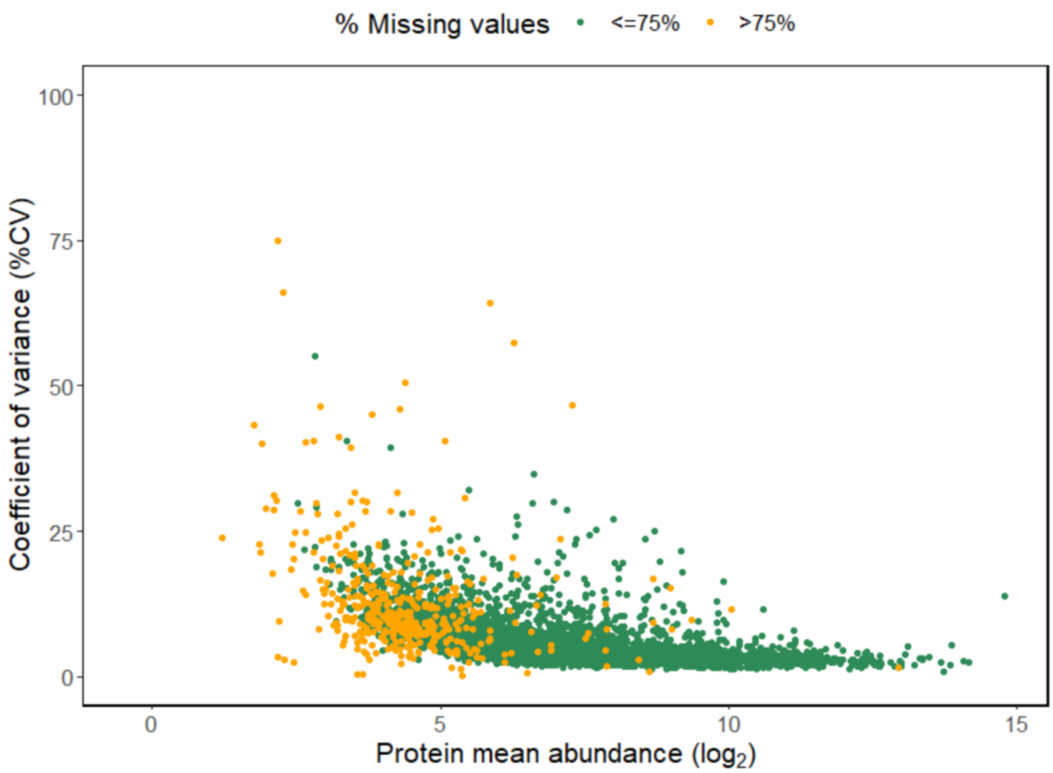

B

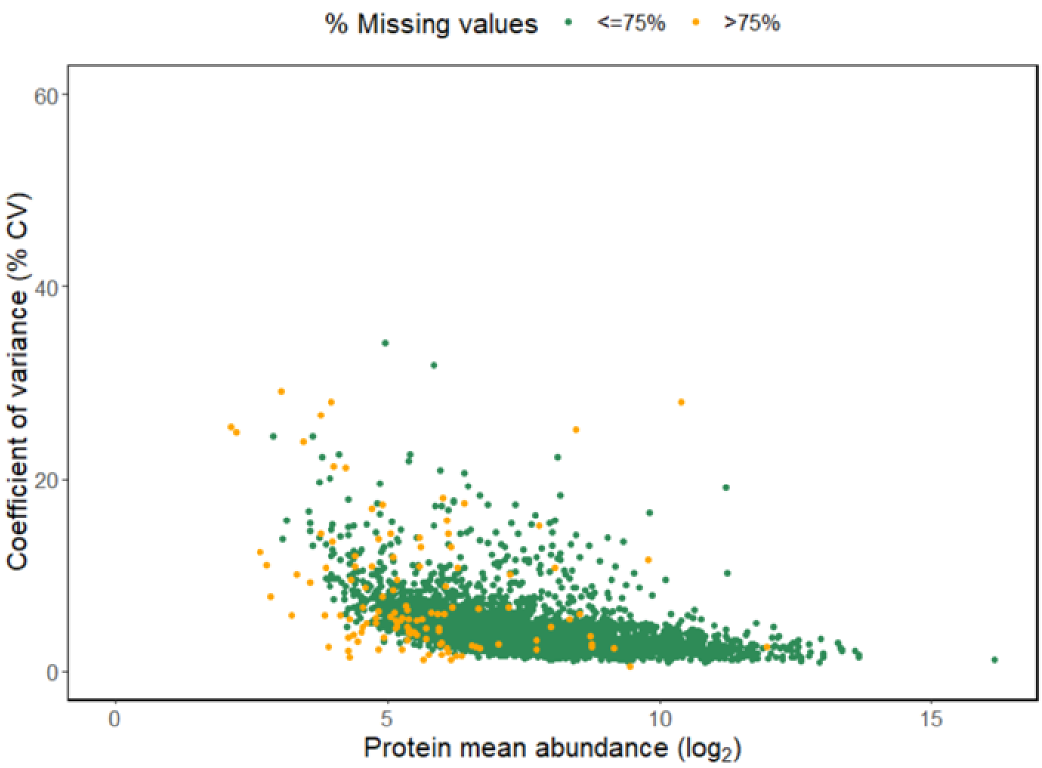

Supplementary Figure S2

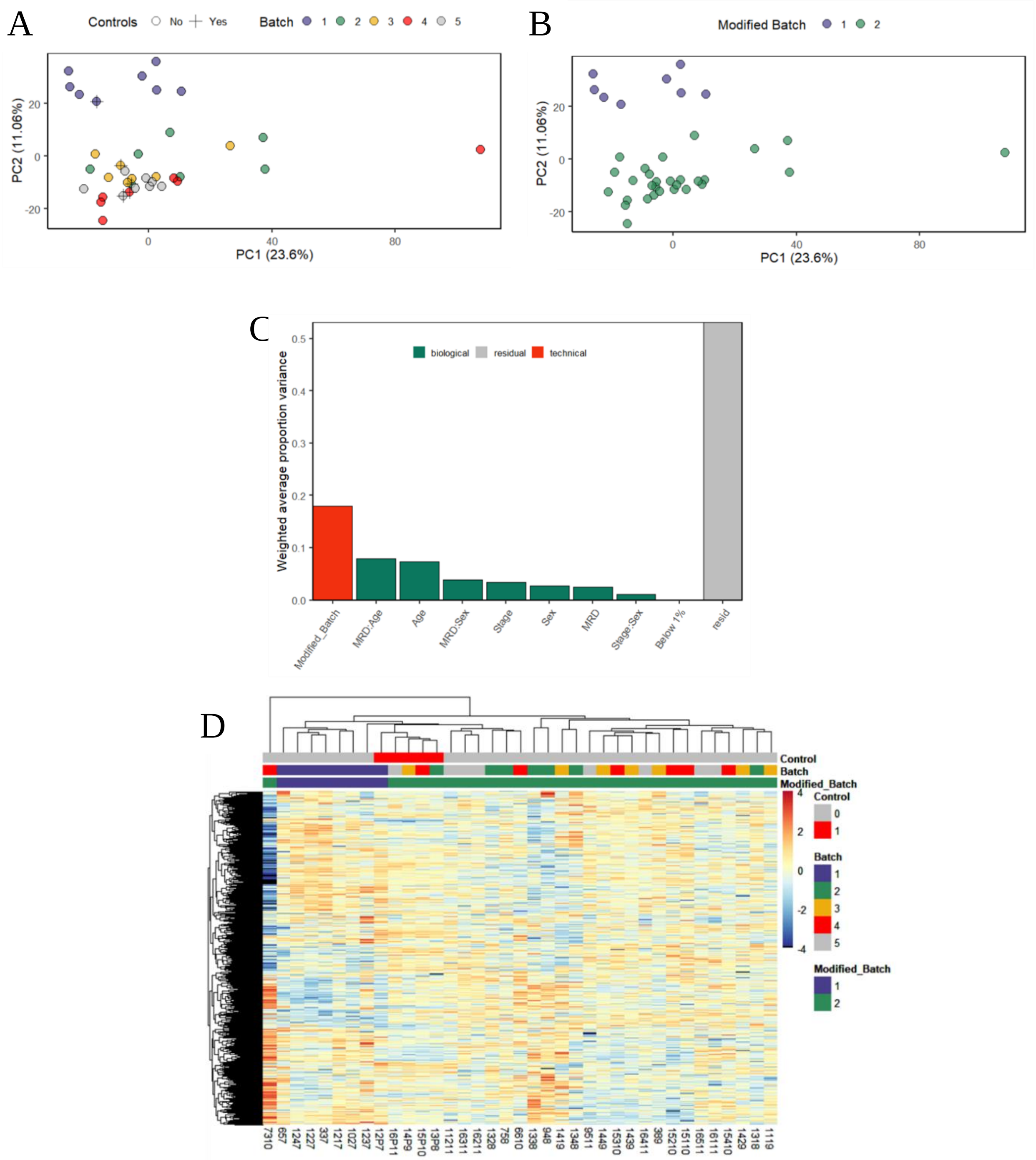

Supplementary Figure S3

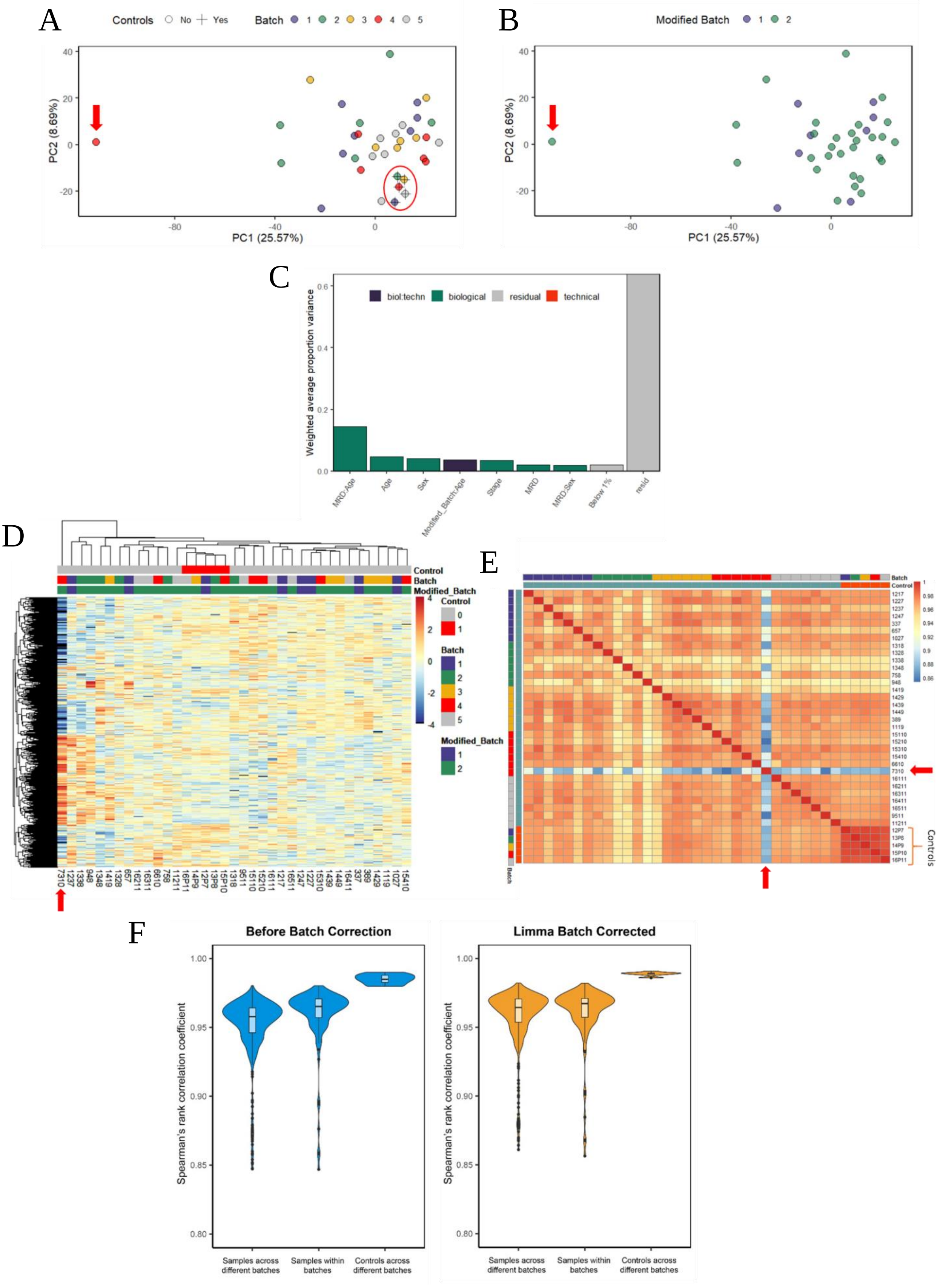

Supplementary Figure S4

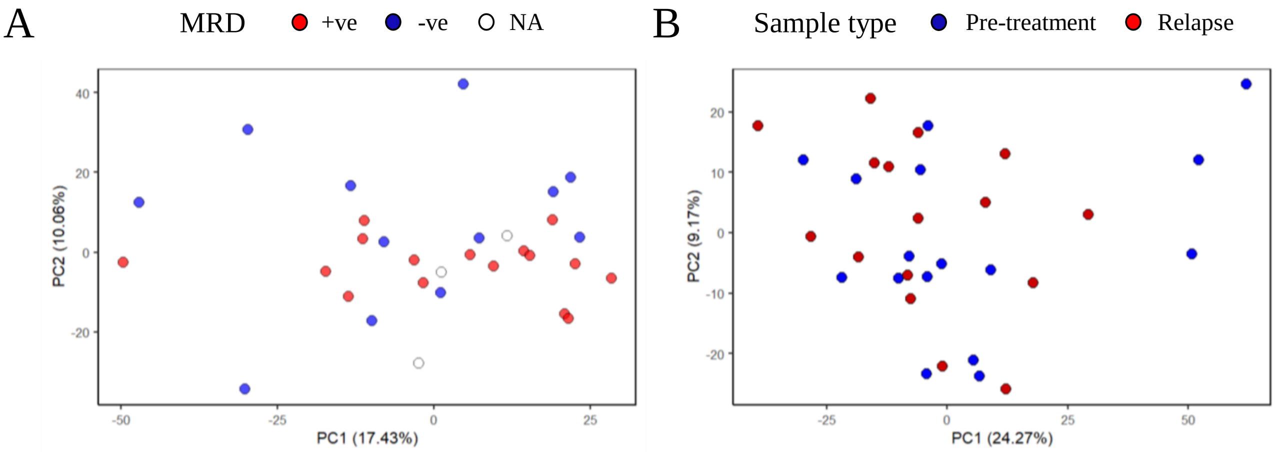

Supplementary Figure S5

A BiP

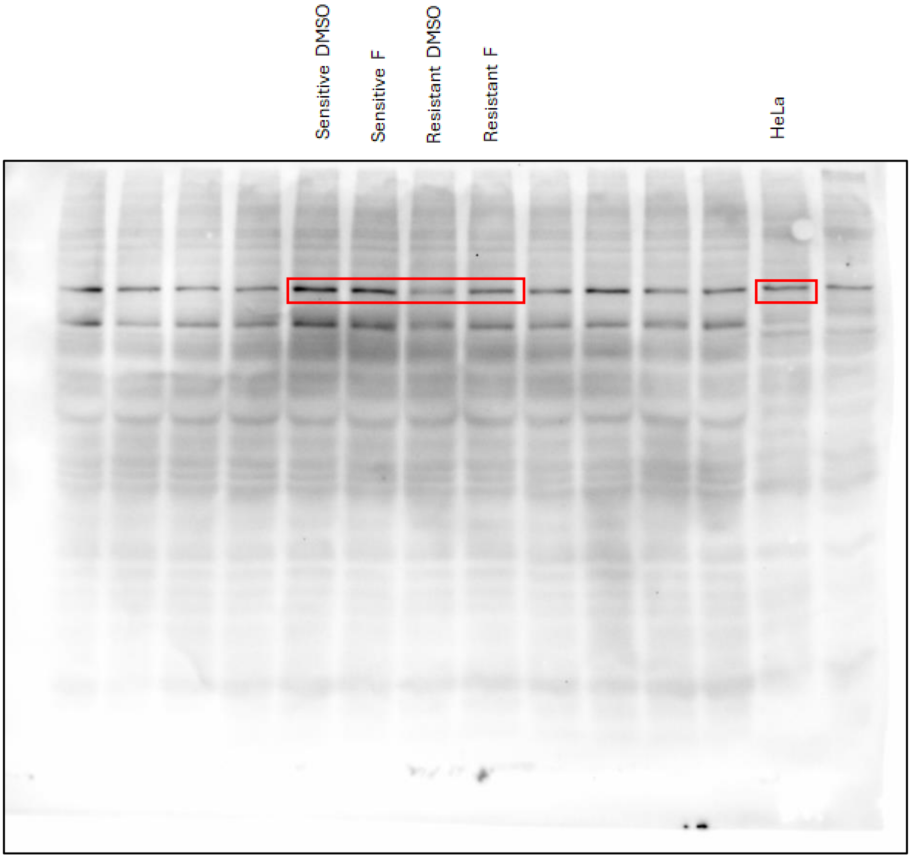

B. PERK

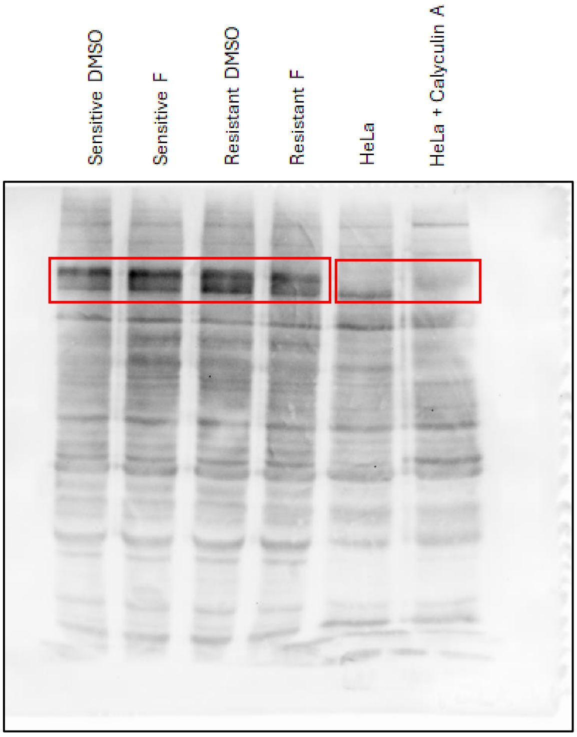

C. p- eIF2α

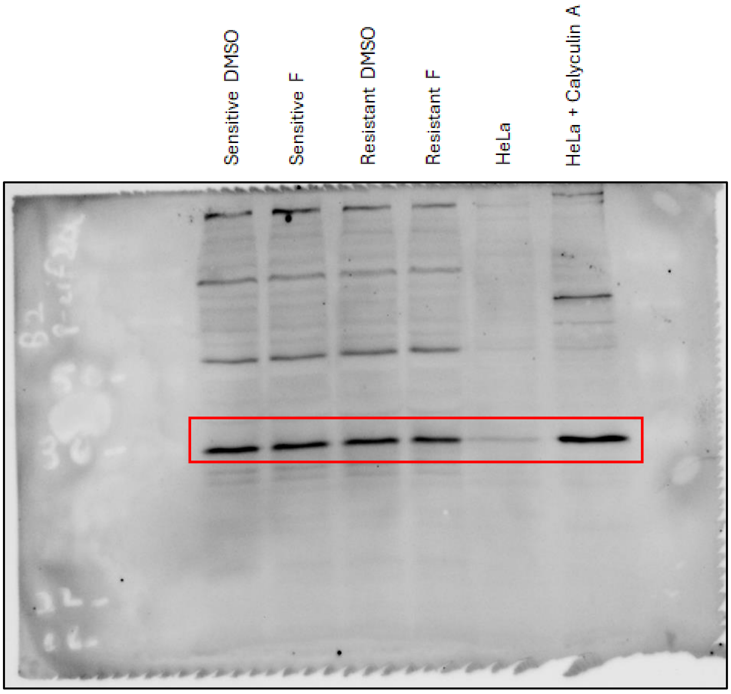

D. Total eIF2α

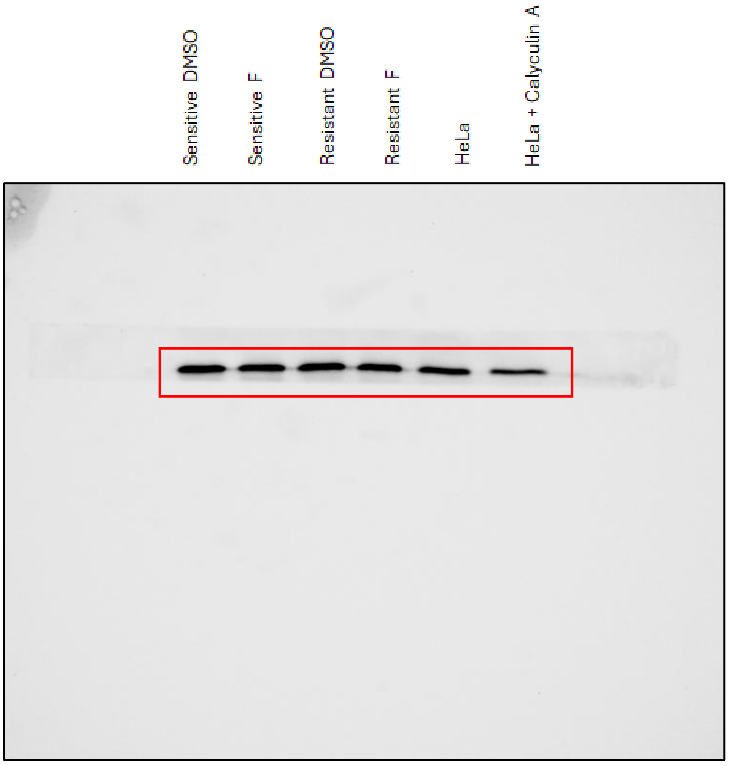

E. Actin

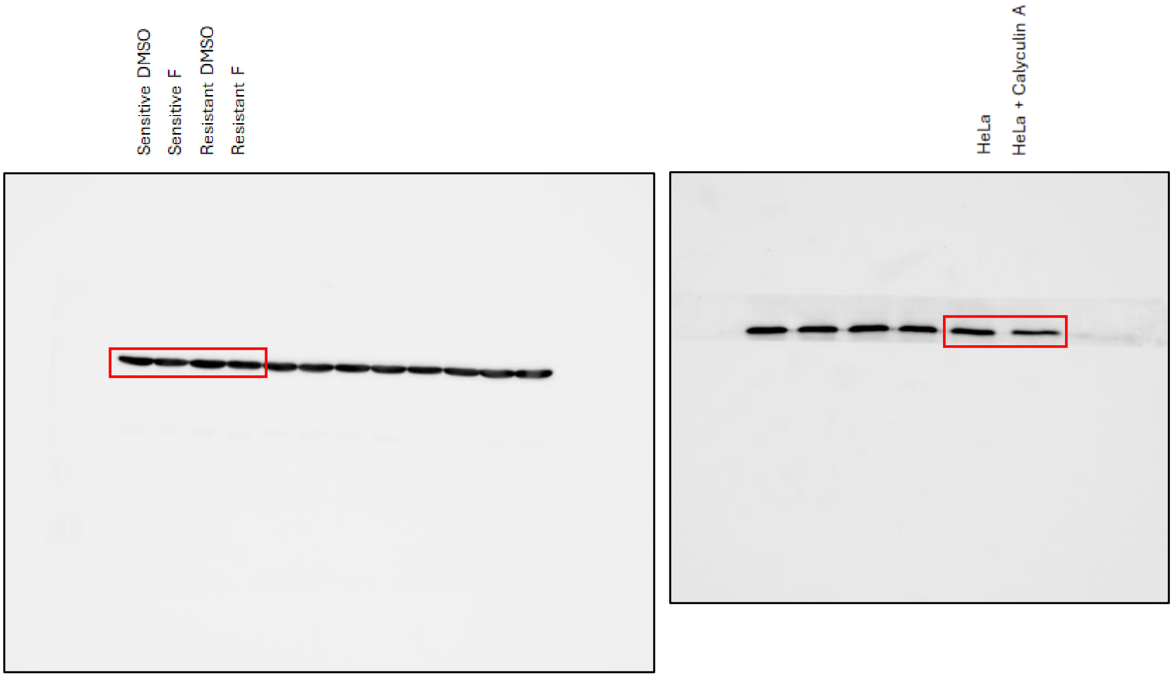
Sensitive DMSO
Sensitive F
Resistant DMSO
Resistant F
HeLa
HeLa + Calyculin A

References

1 R Core Team (2019). R: A language and environment for statistical computing. R Foundation for Statistical Computing, Vienna, Austria. URL https://www.R-project.org/.

2 Wickham, H. ggplot2: Elegant Graphics for Data Analysis. Springer-Verlag New York. (2016).

3 Slowikowski, K. ggrepel: Automatically Position Non-Overlapping Text Labels with 'ggplot2'. R package version 0.8.1. https://CRAN.R-project.org/package=ggrepel. (2019).

4 Leek, J. T. *et al.* sva: Surrogate Variable Analysis. R package version 3.34.0. (2019).

5 Ritchie, M. E. *et al.* limma powers differential expression analyses for RNA-sequencing and microarray studies. *Nucleic Acids Res* **43**, e47 (2015). https://doi.org/10.1093/nar/gkv007

6 Dowle, M. & Srinivasan, A. data.table: Extension of `data.frame`. R package version 1.13.2. https://CRAN.R-project.org/package=data.table. (2020).

7 Cuklina, J. *et al.* Chapter 3: Systematic overview of batch effects in proteomics. Doctoral thesis, ETH Zurich, 2018 https://doi.org/10.3929/ethz-b-000307772.

8 Wickham, H., Franco, D., François, R., Henry, L. & Müller, K. dplyr: A Grammar of Data Manipulation. R package version 1.0.2. https://CRAN.R-project.org/package=dplyr. (2020).

9 Müller, K. & Wickham, H. tibble: Simple Data Frames. R package version 3.0.4. https://CRAN.R-project.org/package=tibble. (2020).

10 Wickham, H. stringr: Simple, Consistent Wrappers for Common String Operations. R package version 1.4.0. https://CRAN.R-project.org/package=stringr. (2019).

11 Wickham, H. & Pedersen, T. L. gtable: Arrange 'Grobs' in Tables. R package version 0.3.0. https://CRAN.R-project.org/package=gtable. (2019).

12 Hastie, T., Tibshirani, R., Narasimhan, B. & Chu, G. pamr: Pam: Prediction Analysis for Microarrays. R package version 1.56.1. https://CRAN.R-project.org/package=pamr. (2019).

13 Kassambara, A. ggpubr: 'ggplot2' Based Publication Ready Plots. R package version 0.2.5. https://CRAN.R-project.org/package=ggpubr. (2020).

14 Auguie, B. gridExtra: Miscellaneous Functions for "Grid" Graphics. R package version 2.3. https://CRAN.R-project.org/package=gridExtra. (2017).

15 Yoshida, K. tableone: Create 'Table 1' to Describe Baseline Characteristics. R package version 0.11.1. https://CRAN.R-project.org/package=tableone. (2020).

16 Dahl, D. B., Scott, D., Roosen, C., Magnusson, A. & Swinton, J. xtable: Export Tables to LaTeX or HTML. R package version 1.8-4. https://CRAN.R-project.org/package=xtable. (2019).

17 Robin, X. *et al.* pROC: an open-source package for R and S+ to analyze and compare ROC curves. *BMC Bioinformatics* **12**, 77 (2011). https://doi.org/10.1186/1471-2105-12-77

18 Heagerty, P. J. & Saha-Chaudhuri, P. survivalROC: Time-dependent ROC curve estimation from censored survival data. R package version 1.0.3. https://CRAN.R-project.org/package=survivalROC. (2013).

19 Therneau, T. A Package for Survival Analysis in R. R package version 3.2-7. https://CRAN.R-project.org/package=survival. (2020).

20 Wickham, H. tidyr: Tidy Messy Data. R package version 1.1.2. https://CRAN.R-project.org/package=tidyr. (2020).
21 Kassambara, A., Kosinski, M. & Biecek, P. survminer: Drawing Survival Curves using 'ggplot2'. R package version 0.4.8. https://CRAN.R-project.org/package=survminer. (2020).
22 Rosen, A. *et al.* Lymphoblastoid cell line with B1 cell characteristics established from a chronic lymphocytic leukemia clone by in vitro EBV infection. *Oncoimmunology* **1**, 18-27 (2012). https://doi.org/10.4161/onci.1.1.18400
23 Stacchini, A. *et al.* MEC1 and MEC2: two new cell lines derived from B-chronic lymphocytic leukaemia in prolymphocytoid transformation. *Leuk Res* **23**, 127-136 (1999). https://doi.org/10.1016/s0145-2126(98)00154-4
24 Zamo, A. *et al.* Establishment of the MAVER-1 cell line, a model for leukemic and aggressive mantle cell lymphoma. *Haematologica* **91**, 40-47 (2006).
